# Changes in pNFH Levels in Cerebrospinal Fluid and Motor Evolution after the Loading Dose with Nusinersen in Different Types of Spinal Muscular Atrophy

**DOI:** 10.3390/medicina59071244

**Published:** 2023-07-04

**Authors:** Mihaela Badina, Gabriel Cristian Bejan, Corina Sporea, Liliana Padure, Andrada Mirea, Madalina-Cristina Leanca, Mihaela Axente, Florin Petru Grigoras, Mihaela Bejan, Elena-Silvia Shelby, Elena Neagu, Daniela Adriana Ion

**Affiliations:** 1 Faculty of Medicine, University of Medicine and Pharmacy “Carol Davila”, 37 Dionisie Lupu Street, 020021 Bucharest, Romania; mihaela.badina@drd.umfcd.ro (M.B.);; 2 National Teaching Center for Children’s Neurorehabilitation “Dr. Nicolae Robanescu”, 44 Dumitru Minca Street, 041408 Bucharest, Romania; 3 Department of Family Medicine, University of Medicine and Pharmacy “Carol Davila”, 8 Eroii Sanitari Bvd., 050474 Bucharest, Romania; 4 Faculty of Midwifery and Nursing, University of Medicine and Pharmacy “Carol Davila”, 37 Dionisie Lupu Street, 020021 Bucharest, Romania; 5 Department of Pathophysiology, National Institute for Infectious Diseases Prof. Dr. Matei Bals, University of Medicine and Pharmacy “Carol Davila”, 1 Calistrat Grozovici Street, 021105 Bucharest, Romania; danielaion7@ymail.com

**Keywords:** spinal muscular atrophy, neurodegenerative disease, neurofilaments, cerebrospinal fluid, pNFH, nusinersen, HFMSE, CHOP INTEND

## Abstract

*Aim and Objectives:* The objective of our retrospective study was to investigate the changes in pNFH levels in cerebrospinal fluid, which is a reliable marker of neuronal damage, after the loading dose of nusinersen in different types of spinal muscular atrophy. *Materials and Methods:* We analyzed the spinal muscular atrophy types, the number of copies of the SMN2 gene, and the progression of the motor status using specific motor function scales in a group of 38 patients with spinal muscular atrophy types 1, 2, and 3. *Results:* We found a significant inverse correlation between pNFH levels and patient age, progress on functional motor scales, and nusinersen administration. Our results also revealed that the neurofilament levels in the cerebrospinal fluid were higher in patients with 2 SMN2 copies than those with more than 2 copies, although the association was not statistically significant due to the abnormal distribution of the values. *Conclusions:* We identified several predictors of favorable evolution under nusinersen treatment, including spinal muscular atrophy type 1, children aged ≤ 30 months, and the presence of only 2 copies of SMN2. Our study provides important insights into the use of pNFH as a biomarker to monitor disease progression and responses to treatment in patients with spinal muscular atrophy.

## 1. Introduction

Neurofilaments (NF) are specific neuronal protein heteropolymers, analyzed over time as markers of neuronal degradation in neurodegenerative diseases [1], which are composed of four subunits: low (NFL), medium (NFM), and high (NFH) (named by size), and α-internexin in the central nervous system or peripherin in the peripheral nervous system [2]. NF are the major neuron cytoskeletal protein, intervening in the increase in thickness of axons, involved in the conduction of nerve impulses [2,3,4], and with activity modulated by various physical and biochemical processes such as phosphorylation, glycosylation, nitration, oxidation, assembly–disassembly, or transport from the place of synthesis to the axonal level [2,3]. NFH, the subunit with the largest size, is ubiquitously found in neurons, with a maximum concentration at the axonal level, and its phosphorylated form (pNFH) is the most resistant to the action of various proteolytic enzymes—an important aspect in choosing it for an analysis as a marker of neuronal degradation [4,5]. Increased NF levels are directly proportional to the degree of neuronal injury and disease progression in various neurodegenerative diseases, such as multiple sclerosis, lateral amyotrophic sclerosis, spinal muscular atrophy, severe burns, Alzheimer’s disease, vascular or frontotemporal dementia, and Parkinson’s disease [6,7,8].

Spinal muscular atrophy (SMA) is a rare neurodegenerative disease that affects approximately 1 in 10,000 newborns, caused by insufficient synthesis of the survival motor neuron (SMN) protein, which occurs in 95% of cases due to a biallelic deletion in the SMN1 gene (5q13) [8,9]. About 80 to 90 percent of the quantity of SMN proteins necessary for the survival of motor neurons is synthesized by the SMN1 gene, while the remaining 10 to 20 percent is produced by the SMN2 gene, a paralogous gene typically present in 2 or more copies located in the same chromosomal region. As the SMN2 gene is the result of the duplication and inversion of SMN1 during evolution, the two genes are almost identical except for 7 base pairs in intron 6, 2 base pairs in intron 7, one base pair in exon 7, and one base pair in exon 8 (cytosine replaces thymine in position 820) [10]. The C to T transition in exon 7 of SMN2 leads to the activation of a splicing situs and the removal of exon 7 of SMN2 by several splicing-promoting proteins. The lack of exon 7, present in approximately 90% of SMN2 transcripts, results in a truncated SMN2 protein that is rapidly degraded by the ubiquitin–proteasome system [10]. Insufficient amounts of SMN protein necessary for normal motor neuron development lead to their progressive loss, resulting in motor acquisition difficulties (gradual muscle weakness accompanied by respiratory failure and associated complications) and the presence of neural components in the CSF and in patient serum [11,12]. SMA is typically divided into five types based on motor development, ranging from type 0 to type 4. In type 0, the symptoms occur prenatally, with decreased fetal movements, joint contractures, and heart defects; the patients require respiratory support at birth, and death occurs before the age of 6 months. In type 1 SMA, the symptoms appear before the age of six months, and such children can sit only with supportive care. Without assisted ventilation, they typically do not survive past 24 months. In type 2 SMA, the symptoms appear before the age of 18 months, and the patients can sit without support and may survive into adulthood. Type 3 SMA is usually diagnosed after the age of 18 months, and all patients achieve walking but lower limb muscle weakness makes them prone to frequent falls, gait disturbances, and eventually loss of ambulation [13]. In type 4 SMA, the symptoms occur after the age of 30 and the patients rarely lose ambulation, and if they do this occurs after the fifth decade of life [13].

The assessment of motor skills is performed using motor functional scales that are adapted to age and are specific to patients with spinal muscular atrophy [14]. The Hammersmith Infant Neurological Examination Section 2 (HINE-2) scale includes eight items, voluntary grasp, ability to kick, head control, rolling, sitting, crawling, standing and walking, with a maximum score of 26. The HINE-2 scale is valid for patients up to 39 months of age. For infants with type 1 SMA, the Children’s Hospital of Philadelphia Infant Test of Neuromuscular Disorders (CHOP INTEND) scale is used in patients aged between 3 months and 21 years to assess the degree of mobility of different body segments, with 16 items and a maximum score of 64 for the correct fulfillment of all requirements [15,16]. The Hammersmith Functional Motor Scale Expanded (HFMSE) is a scale developed specifically for SMA patients, which investigates a patient’s ability to perform various activities and has a total score of 66 points. The HFMSE is used in patients aged between 2 and 45 years. The Revised Upper Limb Module for Spinal Muscular Atrophy (RULM) investigates the upper limb function of ambulatory and non-ambulatory SMA patients over 2 years old [14], with a maximum score of 37 points, and is used in patients aged between 2 and 52 years.

In 2016, the U.S. Food and Drug Administration (FDA) approved Spinraza (nusinersen), the first drug for SMA, a modified antisense oligonucleotide that acts on the expression of the SMN2 gene, leading to the production of a full-length SMN2 transcript [17]. This transcript will produce the complete SMN protein necessary for the survival of the motor neurons, thereby preventing their destruction and interrupting the mechanism underlying the appearance of the disease. The treatment, with a recommended dosage of 5 mL (12 mg) of Spinraza administered intrathecally, consists of a loading phase, which involves four doses administered on days 0, 14, 28, and 63, followed by maintenance phases occurring every four months [15].

We think that the initiation period can be considered as having the greatest impact on the clinical condition of the patients, with reductions until the disappearance of the destruction of the motor neurons by ensuring the sufficient amount of SMN necessary for their survival. The evolution of the pNFH level during this period is mostly on reducing the damage to the motor neurons due to the treatment.

The aim of this study was to analyze the changes in pNFH levels in cerebrospinal fluid after the administration of the loading treatment with nusinersen compared to the initial level of this biomarker, at different ages, with different phenotypes, and in different types of SMA. Additionally, we investigated the relationships between the results obtained on motor function evaluation scales and the evolution of the pNFH levels in cerebrospinal fluid as an expression of the rescue of motor neurons. 

## 2. Materials and Methods

In this monocentric retrospective study, we examined the level of pNFH neurofilaments in cerebrospinal fluid during the first six months of treatment, which corresponds to the administration of loading doses with nusinersen. The analysis was conducted by collecting two samples, before the initiation of treatment (first sample) and six months after the initiation, at the end of the loading period, just before the administration of the first dose of maintenance treatment (second sample). The results were analyzed based on the age, the SMA type, the number of SMN2 copies, and the evolution on specific motor functional scales.

We followed the evolution of 38 genetically confirmed patients diagnosed with SMA who were accepted into the National Treatment Program with Nusinersen between October 2018 and July 2021. To be eligible for the study, the patients had to be under 18 years of age, have bi-allelic pathogenic or likely pathogenic mutations of the SMN1 gene, possess a minimum of two copies of the SMN2 gene, and have a general condition unaffected by any additional acute disease. The exclusion criteria for the study were as follows: the existence of another disease-modifying treatment and the presence of other known diseases that increase the level of neurofilaments, temporary diseases that can influence motor performance, recent acute or chronic inflammatory diseases, other diseases that evolve with acute or chronic neuromotor degeneration, or cerebral or spinal disorders that would interfere with performing a lumbar puncture or influence the circulation of cerebrospinal fluid [16].

The study was approved by the Ethics Committee of the National Teaching Center for Children’s Neurorehabilitation “Dr. Nicolae Robanescu” (agreement numbers 7464/01.10.2018 and 8818/16.12.2020), and all patients had the consent of the authorized legal owners to participate, in accordance with local regulations and the World Medical Association Declaration of Helsinki, revised in 2000, Edinburgh. The legal caregivers of the participants were informed about the purposes of the use of their data, and all patient data were correctly anonymized.

The clinical and laboratory assessments, as well as the treatment, were carried out by specialized, trained, and certified personnel, including pediatricians and pediatric neurologists for clinical parameters, physical therapists for functional parameters, and laboratory doctors for biological parameters. All evaluations were made by the same person to avoid the risk of bias in the analysis and the interpretation of the data. After the neurological and pediatric evaluations, qualified staff specialized in assessing children with SMA at the Dr. Nicolae Robanescu National University Center for Children’s Neurorehabilitation evaluated the patients on mobility and physical ability assessment scales recommended according to age and locomotor status for children with SMA [11], just before the administration of nusinersen. For the study, we considered the CHOP INTEND scale, specifically created for children with SMA between 1.4 and 38 months, scored between 0 and 64 points for the 16-item scale with four levels of response complexity, and the HFMSE scale, which is used for patients over 24 months and has 33 operations scored between 0 and 2 points, with a maximum score of 66 points.

Before the administration of nusinersen, cerebrospinal fluid samples were collected in aseptic conditions through lumbar puncture in an amount of approximately 5 mL according to the administration protocol of the drug [18]. The collected samples were divided into two sterile samples, one analyzed before preservation and the other preserved at a temperature lower than −20 °C, according to the instructions of the analysis kit manufacturer. The cerebrospinal fluid samples were analyzed before preservation treatment from the following perspectives: macroscopic appearance, ionogram, and cellularity. The macroscopic appearance was qualitatively assessed based on turbidity (clear appearance—0—McFarland standard; cloudy—any other value) and color, which was evaluated using a four-step visual scale: 0—colorless liquid; 1—liquid with a slight shade of red; 2—liquid with an intense shade of red; 3—liquid with a hemorrhagic appearance. For the ionogram, we used the potentiometric method with the I Smart-30 Pro ionometer (i-Sens, Inc., Seoul, Republic of Korea). The cellularity was determined using the Bürker–Türk (Fischer Scientific Company L.l.c., P.O. Box 1768, Pittsburgh, PA 15275, USA) counting chamber and optical microscopy with a Nikon Eclipse E 100/300 microscope (Nikon, Minato City, Tokyo, Japan).

The demographic, anthropometric, motor development milestone achievement, clinical, and laboratory data of the participants were collected from the patient observation sheets and the hospital computer program.

The statistical analyses were conducted using IBM SPSS Statistics 24 and Microsoft Excel 2021 software. The continuous variables were reported as means ± SDs (standard deviations) if variables were uniform distributed or as medians (IQR, interquartile range) if the variables were abnormally distributed. In cases of continuous variables with a uniform distribution, we used a paired *t*-test for comparisons of the central tendency of the baseline characteristics and a Wilcoxon signed ranks test for continuous variables with an abnormal distribution. The normality of distributions was assessed using the D’Agostino–Pearson test [19]. The Mann–Whitney test [20,21] was used to compare groups, while paired *t*-tests [22,23,24] or Wilcoxon signed-rank tests [25,26] were used to compare variables over time. The non-parametric Spearman correlation coefficient [27] was used to assess associations between variables with an anomalous distribution. A *p*-value less than 0.05 was considered statistically significant, and all statistical tests were two-tailed [28,29]. We used a linear regression equation to predict the evolution of the pNFH levels after treatment relative to the baseline pNFH level.

## 3. Results

Of the patients included in the study, 18.42% were diagnosed with SMA type 1 and all had 2 SMN2 copies. SMA type 2 was present in 52.63% of the patients, of which 20% had 2 SMN2 copies, 75% had 3 SMN2 copies, and 5% had 4 SMN2 copies. SMA type 3 was present in 28.95% of the patients, of which 27.27% had 2 SMN2 copies and the remaining 72.73% had 3 SMN2 copies, as shown in Table 1.

**Figure 1:**
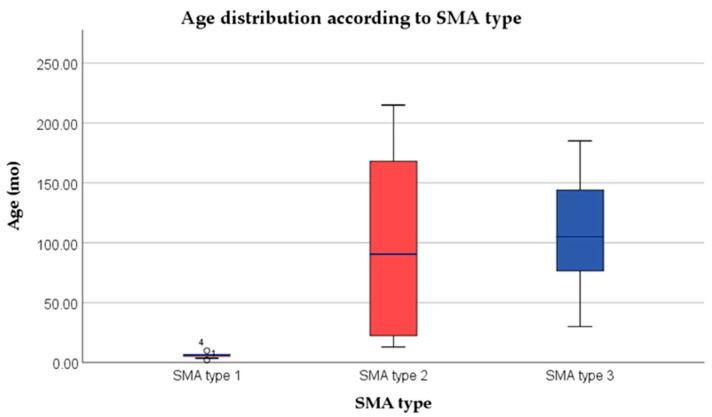
Age distribution at the initiation of the treatment according to SMA type.

There were significant differences in the age at the onset of treatment among the SMA types in the study group, with mean ages ranging from 2.07 to 215 months, with type 1 patients starting treatment at a much younger age than those with types 2 and 3 (as shown in Figure 1).

The motor scale results are presented as relative values to the maximum possible score. These relative values are more relevant in terms of progression and allow for a comparison between the two types of scales (refer to Table 2).

The pNFH level (ng/dL) was determined using the ELISA technique, according to the instructions of the manufacturer of the reagent kit, EUROIMMUN Medizinische Labordiagnostika AG (Seekamp 31, 23560 Lübeck, Germany), with the help of the ELISA BIORAD 3100 PSC microplate reader (Bio-Rad Laboratories, 1000 Alfred Nobel Drive, Hercules, CA 94547, USA) (Table 3).

The range of pNFH neurofilament levels in the cerebrospinal fluid before treatment was quite wide, ranging from 0.035 ng/dL in a 104-month-old patient with 3 SMN2 copies, diagnosed with SMA type 2, to 3.321 ng/dL in a 3.63-months-old patient diagnosed with SMA type 1 with 2 SMN2 copies, the patient with one of the most severe forms of the disease.

No statistically significant correlations were observed between the pNFH level and macroscopic appearance, chlorine ion concentration, or cellularity, neither before the initiation of treatment nor after 6 months of treatment.

**Figure 2:**
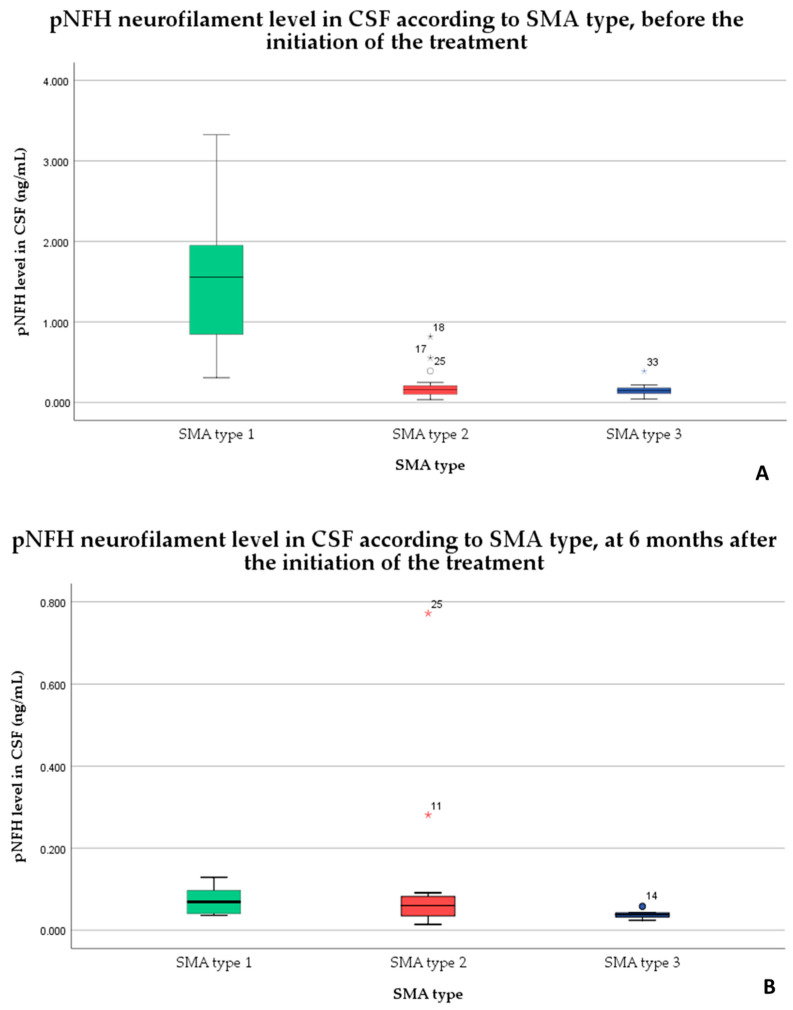
The pNFH levels in cerebrospinal fluid before (**A**) and 6 months after the initiation of the treatment (**B**) according to SMA type.

We identified 12 statistically significant correlations between the analyzed variables. Thus, the pNFH neurofilament level before the initiation of treatment (pNFHi) was moderately correlated with the patients’ age (*p* < 0.0001, r = −0.588) and SMA type (*p* < 0.001, r = −0.528). The pNFH neurofilament level after 6 months of treatment (pNFHf) was moderately correlated with the pNFHi (*p* < 0.05, r = 0.409) and weakly correlated with the SMA type (*p* < 0.05, r = −0.394) and motor scores before the initiation of treatment (*p* < 0.05, r = −0.363). The motor scores after 6 months of treatment were strongly correlated with the motor scores before the initiation of treatment (*p* < 0.0001, r = 0.765), moderately correlated with the patients’ age (*p* < 0.05, r = −0.438), and weakly correlated with the number of SMN2 copies (*p* < 0.05, r = −0.337). The SMA types were moderately correlated with the motor scores before the initiation (*p* < 0.05, r = 0.407) and the patients’ age (*p* < 0.0001, r = 0.553) and weakly correlated with the number of SMN2 copies (*p* < 0.05, r = 0.390). The patients’ age was moderately correlated with the number of SMN2 copies (*p* < 0.0001, r = 0.541). The distribution of pNFH neurofilament levels in cerebrospinal fluid, before and 6 months after the initiation of the treatment, according to SMA type, is shown in Figure 2.

There has been identified 7 unusual values in the levels of cerebrospinal fluid pNFH in relation to the different types of SMA and the timing of the treatment. Before the treatment started, there were 4 outliers: three for SMA type 2, which included one outlier that was classified as an “out value” (represented by a circle) and two outliers classified as “extreme values” (represented by stars). Additionally, there was one extreme value outlier for SMA type 3. After 6 months of treatment, there were 3 outliers: two extreme values for SMA type 2 and one out value for SMA type 3. The out values are moderately far from the central range of the data, respectively more than 1.5 interquartile range (IQR’s) but less than 3 IQR’s from the upper and lower boundaries of the boxplot, which represent the 75th percentile (Q3) and the 25th percentile (Q1). An outlier is considered to be “out” if it falls above 1.5 times the IQR (the range between Q3 and Q1) but below 3 times the IQR from the end of the box. The extreme value refers to the values that are even farther from the central range of the data, and are considered outliers if they exceed 3 times the IQR. 

**Figure 3:**
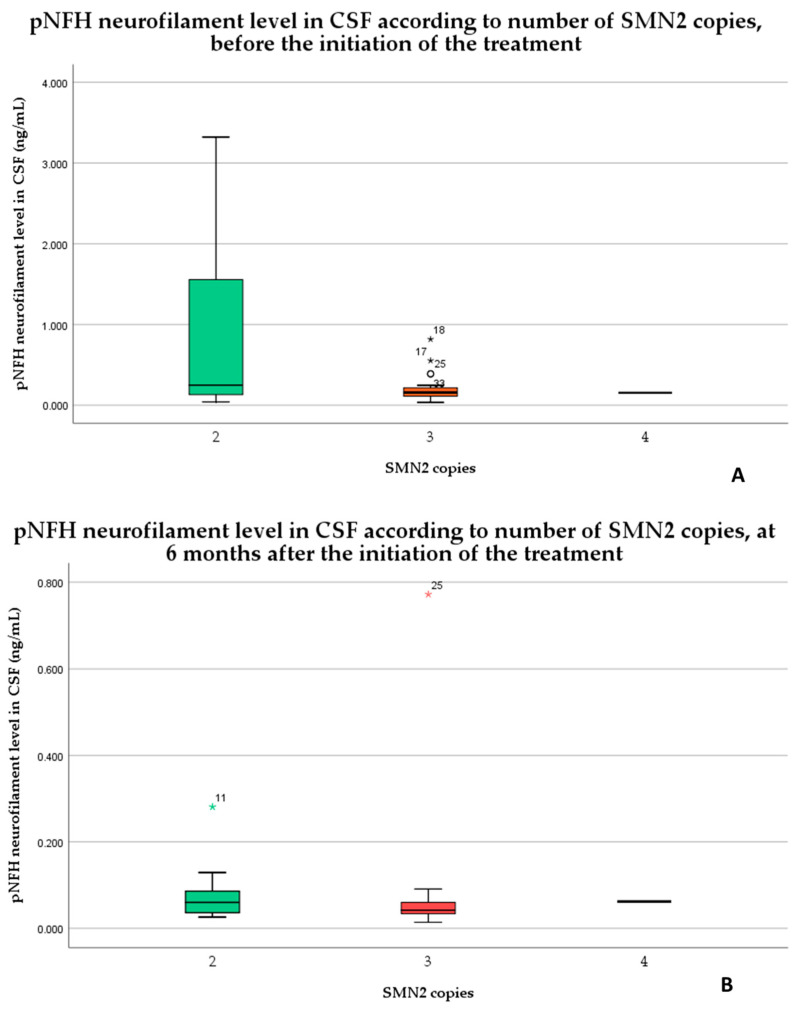
The pNFH levels in the cerebrospinal fluid before (**A**) and 6 months after the initiation of the treatment (**B**) according to the SMN2 copies.

The patients with 2 SMN2 copies had a higher level of neurofilaments in the cerebrospinal fluid compared to patients with more than 2 SMN2 copies (without statistically significance, *p* = 0.08), as shown in the graph provided in Figure 3.

There has been identified 5 unusual values in the levels of cerebrospinal fluid pNFH in relation to the different numbers of SMN2 copies and the timing of the treatment. Before the treatment started, there were three outliers for 3 SMN2 copies, which included one outlier that was classified as an “out value” (represented by a circle) and two outliers classified as “extreme values” (represented by stars). After 6 months of treatment, there were two extreme values, one for 2 SMN2 copies and one for 3 SMN2 copies. 

The difference between the pNFH level (ng/mL) before treatment and after 6 months of treatment was statistically significant in terms of the decrease in the amount of neurofilaments in the cerebrospinal fluid after treatment (the 4 loading doses), according to the paired *t*-test for SMA types 1 and 3 and the Wilcoxon signed ranks test for SMA type 2:

- SMA type 1: *t*(6) = 3.848, *p* = 0.008;
- SMA type 2: z = −2.501, *p* = 0.012;
- SMA type 3: *t*(10) = 4.088, *p* = 0.002.

Regression equations for the pNFH levels after treatment (pNFH2) depending on the pNFH levels before treatment (pNFH1) for each type of SMA are shown in Table 4.

**Figure 4:**
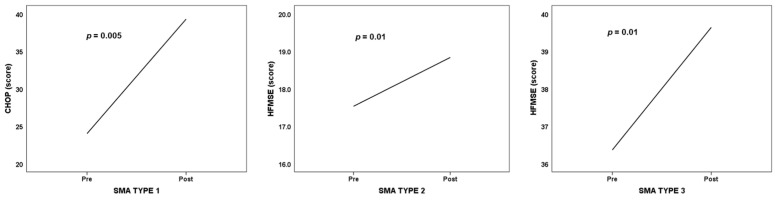
Motor function evolution on motor scales according to SMA type.

Analyzing the motor function evolution after the nusinersen treatment, based on the motor scale assessment scores and using a paired samples test, we found statistically significant improvement in all types of SMA, as shown in Figure 4:

- CHOP INTEND (SMA type 1): *t*(6) = 4.398, *p* = 0.005;
- HFMSE (SMA type 2): *t*(17) = 2.832, *p* = 0.012;
- HFMSE (SMA type 3): *t*(10) = 3.184, *p* = 0.01.

For the studied group, from the point of view of evolution on the functional motor scales, the increase in score percentages on the CHOP INTEND and HFMSE scales 6 months from the initiation of nusinersen treatment compared to the initial values relative to the maximum value of the motor scales, 28 patients (73.7%) out of the 38 patients showed a positive evolution from the point of view of increasing the relative score obtained on the motor function evaluation scales 6 months from the initiation of the treatment, 10.5% (4 patients) showed no changes in scores, and 6 patients (15.8%) showed a decrease in motor evolution. These relative percentage increases were between a minimum of –100 and a maximum of 262.5, with an average of 30.83% (SD = 61.5%), a median of 13.92% (IQR = 42.7%), and a *p*-value of 0.001.

## 4. Discussion

In terms of the demographic and clinical characteristics, the study included a diverse group of patients with varying ages and types of SMA, as well as different numbers of SMN2 copies. This allowed for a comprehensive analysis of the relationship between these factors and the level of pNFH in the cerebrospinal fluid, as well as the effectiveness of the nusinersen treatment for motor function. It is important to emphasize that SMA is a rare disease, and the number of patients included in our study is significant for this rare genetic condition. 

The wide range of pNFH values observed in the study is also notable, with the highest levels of pNFH being determined before the initiation of treatment, in the most serious forms of the disease, and in patients with the lowest scores on the functional motor scales, similar to the results of other studies in the literature [30,31,32]. 

Like other studies, the findings show that nusinersen treatment can lead to improvements in motor function and a decrease in pNFH levels in pediatric patients with SMA [33]. The treatment efficiency is proven by the slowdown or stopping of the progression of the disease (based on clinical symptoms) and by changes of the scores obtained by patients on the motor scales. The correlation between the level of pNFH in the cerebrospinal fluid and the scores obtained on the motor scales for SMA in the first 6 months of treatment was negative; that is, the decrease in the level of pNFH during treatment evolved with increasing MFM scores, although the correlation was statistically significant only in the case of type 2 SMA. Considering the dose of nusinersen administered in accordance with the drug protocol indicated by the manufacturer compensates for the lack of SMN needed and covers the needs for SMN for the survival of motor neurons in order to compare the effectiveness of the treatment while taking into account the different functional motor scales applied to the patients; the percentage variation of the parameters (pNFH level and relative score on the MFM scales) was more relevant than the absolute difference of these values. The yield of changes is represented by the percentage of the difference between the initial level of neurofilaments compared to the level obtained after 6 months of treatment, and for the motor scales this is represented by the percentage of the difference between the relative value of the score 6 months after the initiation of treatment compared to the initial relative value (relative value compared to the maximum value of the scale).

Based on the non-parametric distribution of the relative percentage increases in the MFM scale scores, we identified a cut-off value of 13.92% for better motor function improvement after 6 months of nusinersen treatment. Upon the analysis of patients with values above the cut-off, we identified the factors that predicted a favorable outcome under nusinersen treatment: diagnosis of type 1 SMA, age of children ≤ 30 months, 2 copies of SMN2 gene, a baseline pNFH neurofilament level > 0.18 ng/mL, a difference between the final and initial pNFH values greater than 0.15 ng/mL, and a percentage decrease in neurofilaments (pNFH) in the cerebrospinal fluid after 6 months of nusinersen treatment of more than 85.99%.

We consider that the results of the study are limited by the possibility of the existence of some additional factors, independent of the underlying disease, which could interfere both with the level of pNFH and with the performances on the motor scales, with special roles played by the degree of neuromuscular degradation at the time of treatment initiation and the individual ability to follow physical therapy. Changes in the level of pNFH over time due to treatment with nusinersen can be influenced by the occurrence of episodes of acute diseases between the administered doses, episodes that can evolve with neuronal destruction on the one hand but can also influence the evolution on the functional motor scales by limiting the temporary motor capacity and compliance with the physical therapy program.

## 5. Conclusions

With the deepening of knowledge about SMA, the evolution of the technology in the field of genetics, the possibility of an early diagnosis, and especially the approval of some disease-modifying treatments, the classification of the types of this rare disease based on functional criteria has become controversial. Being a recently approved treatment, for these patients, the time of initiation of the treatment is not related to the onset of symptoms or the time of diagnosis. 

After six months of receiving nusinersen treatment, the motor function of most of the patients included in the study showed an improvement, as revealed by an increase in their scores on the motor function scales, while a few of the patients did not show any changes in their scores or experienced a decrease. These findings suggest that the majority of patients experienced a positive response to the nusinersen treatment in terms of increased motor function scores and decreased levels and percentage variation of pNFH in the cerebrospinal fluid. 

The treatment with nusinersen has been shown to have a high clinical success rate in patients with spinal muscular atrophy, especially for serious forms of the disease, with the treatment initiated as early as possible and with a value as high as possible of the level of pNFH in the CSF at the initiation of the treatment. However, it is important to continue monitoring these patients over time and also study any other groups of patients to check in detail the importance of each of these predictive factors to determine the effectiveness of the treatment in terms of the clinical and motor recovery for a better understanding of the disease and its treatment, and to potentially improve the outcomes for patients with spinal muscular atrophy.

## Figures and Tables

**Table 1:** Characteristics of the group of study participants.

	SMA				No. of SMN2 Copies	
	All types	Type 1	Type 2	Type 3	2 copies	>2 copies
No	38	7	20	11	14	24
Sex—male	19	4	11	4	5	14
Age (months)						
Min ÷ max	2.07 ÷ 215.00	2.07 ÷ 9.87	13.00 ÷ 215.00	30.00 ÷ 185.00	2.07 ÷ 131.00	13.00 ÷ 215.00
(Mean)	(82.13)	(5.88)	(96.05)	(105.36)	(38.16)	(107.79)
SMN copies						
No						
2 copies	14	7	4	3		
3 copies	23	0	15	8		
4 copies	1	0	1	0		

**Table 2:** Relative values of the Motor Function Measure (MFM) scale to the maximum possible score (mean ± SD for variables with normal distribution and IQR for variables with non-normal distribution).

	SMA				No. of SMN2 Copies	
	All types	Type 1	Type 2	Type 3	2 copies	>2 copies
MFM—pre mean ± SD	28.03%	37.50% ± 8.85%	23.83% ± 3.34%	55.10% ± 5.92%	26.14%	32.89% ± 4.07%
MFM—post mean ± SD	43.07% ± 4.18%	61.38% ± 6.42%	27.32% ± 4.39%	60.06% ± 6.73%	55.11% ± 6.58%	36.05% ± 4.93%

**Table 3:** Relative values of pNFH levels in cerebrospinal fluid depending on the SMA type and number of SMN2 copies (mean ± SD for variables with normal distribution and IQR for variables with non-normal distribution).

	SMA				No. of SMN2 Copies	
	All types	Type 1	Type 2	Type 3	2 copies	>2 copies
pNFH—pre mean ± SD median (IQR)	0.18	1.54 ± 0.39	0.16	0.16 ± 0.03	0.25	0.16
pNFH—post mean ± SD median (IQR)	0.04	0.07 ± 0.01	0.06	0.04 ± 0.003	0.06	0.04

**Table 4:** Regression equation of the pNFH levels after treatment.

SMA Type	Regression Equation	*p*-Value
Type 1	pNFH2 = 0.054 + 0.012 × pNFH1	0.46
Type 2	pNFH2 = 0.063 + 0.176 × pNFH1	0.40
Type 3	pNFH2 = 0.039 − 0.01 × pNFH1	0.78

## Data Availability

The corresponding authors can provide the data described in this study upon request.
